# Pressure ulcer risk in patients undergoing cardiovascular surgery and their occurrence within 24 hours of the operation

**DOI:** 10.1590/1980-220X-REEUSP-2025-0081en

**Published:** 2025-08-04

**Authors:** Zeynep Gürer, Neriman Akansel, Nail Kahraman

**Affiliations:** 1 Health Sciences University, Bursa City Hospital, Operating Room Staff Nurse, Bursa, Türkiye.; 2 Bursa Uludag University, Faculty of Health Sciences, Department of Surgical Nursing, Bursa, Türkiye.; 3 Health Sciences University, Bursa City Hospital, Department of Cardiovascular Surgery, Bursa, Türkiye.

**Keywords:** General Surgery, Nursing Care, Operating Rooms, Pressure Ulcer, Cirurgia geral, Cuidados de Enfermagem, Salas Cirúrgicas, Úlcera por Pressão

## Abstract

**Objective:**

This study aimed to determine the risk of pressure ulcer (PrU) in cardiovascular surgery (CVS) patients and assess their occurrence within 24 hours of the operation.

**Method:**

The study was conducted with CVS patients (n = 200) meeting the inclusion criteria. The data was collected using the Patient Introduction Form and the Turkish version of the 3S Intraoperative Pressure Injury Risk Assessment Scale (3S IPIRAS). PrU development was assessed within 24 hours of the operation.

**Results:**

The mean age of patients was 63.61, and 32.0% were under 60 years old. Sixty-five percent of the patients were male, and 40.5% were overweight. Being female and the body mass index (BMI) score influenced the risk of PrU development. Some of the patients (14%) developed a Stage I PrU within 24 hours of the surgery.

**Conclusion:**

CVS patients are at risk of developing PrU, and the occurrence of PrU was high in this study. Preventive measures in the perioperative period and increasing awareness of PrU among healthcare personnel are essential.

## RESUMO

**Objetivo:**

Este estudo tem como objetivo determinar o risco de úlceras por pressão (UPP) em pacientes submetidos à cirurgia cardiovascular (CCV) e avaliar sua ocorrência nas primeiras 24 horas após a operação.

**Método:**

O estudo foi realizado com pacientes de CCV (n = 200) que atendiam aos critérios de inclusão. Os dados foram coletados por meio do Formulário de Apresentação do Paciente e da versão turca da Escala de Avaliação de Risco de Lesão por Pressão Intraoperatória 3S (3S IPIRAS). O desenvolvimento de UPP foi avaliado nas primeiras 24 horas após a cirurgia.

**Resultados:**

A idade média dos pacientes foi de 63,61 anos, e 32,0% tinham menos de 60 anos. Sessenta e cinco por cento dos pacientes eram do sexo masculino, e 40,5% estavam com sobrepeso. O sexo feminino e o IMC influenciaram o risco de desenvolvimento de UPP. Alguns pacientes (14%) desenvolveram uma UPP de Estágio I nas primeiras 24 horas após a cirurgia.

**Conclusão:**

Pacientes submetidos à CCV estão em risco de desenvolver UPP, e a ocorrência foi alta neste estudo. Medidas preventivas no período perioperatório para aumentar a conscientização entre os profissionais de saúde são essenciais.

## INTRODUCTION

Pressure ulcers (PrUs) are localized ruptures usually found on bony prominences; they are caused by exposure to external pressure on the skin or subcutaneous tissue^(1)^. Surgical patients are at risk of developing PrU throughout the entire perioperative process, which includes preoperative, intraoperative, and post-operative care. Although patient-related factors (e.g. chronic disease, age, being male, obesity, malnutrition, condition of the skin, anemia) feature in the development of PrUs, operating room (OR) environment-related incidents (e.g. remaining in the same position for a long time, duration of surgery, anesthesia, use of vasopressors, blood loss, hypothermia, medical equipment, friction) are undeniable^(2,3,4,5,6)^. In cardiovascular surgery (CVS) specifically, environmental factors can provoke the development of PrU^(4,5)^. Device-related PrUs at bone protuberances, such as the cheek, chest, heel, and auricle, are common in surgical patients^(5)^.

In one study, the incidence of PrUs in adult patients undergoing cardiovascular surgery was 19.86%^(7)^. The percentages of PrUs related to the surgical process vary from 1.3% to 17.6%^(8,9)^. In a meta-analysis conducted to determine the incidence of PrUs in different countries, including Turkey, the incidence was as high as 12%^(9)^. Stage-1 pressure injuries were detected in 6.7% of the patients in another study, and the occurrence of PrUs was found to harm the quality of care, threaten patient safety, extend the length of hospital stay, and increase the cost of care^(10)^. However, fully eliminating OR-related PrUs is impossible due to their multiple etiologies and the challenges of assessing risk factors^(11)^.

The PrU Prevention and Treatment Quick Reference Guideline emphasizes that PrU risk assessment should be performed every time the patient’s condition changes, before, during, and after surgery^(12)^. Although many scales are available for assessing the risk or PrU (the Norton Scale, the Braden Scale, the Waterlow Scale, the Gosnell Scale, and the Knoll Scale), none are adequately able to predict PrU risk in patients undergoing surgery. One study done using the Munro PrU Risk Assessment Scale reported that this scale is effective in predicting PrUs in the post-operative period in patients who have received general anesthesia^(13)^. Randomized controlled studies focusing on the perioperative process require a high level of evidence for the occurrence of PrUs in CVS patients^(14,15)^.

The use of the 3S Intraoperative Pressure Injury Risk Assessment Scale (3S IPIRAS) is recommended for the evaluation of patients with a high risk of PrUs in the OR. In the Turkish validation study of this scale with various surgical patients (n = 187), eight patients assessed with risk of developing PrU in the OR experienced stage one PrU formation after surgery^(11)^. Surgical nurses are crucial to patient safety throughout the perioperative process^(2)^. Evidence-based practices and case-specific interventions in the OR to prevent undesirable outcomes such as PrUs are essential^(3,6,16)^.

Research on assessing the risk of PrU in the OR environment is limited, and this also applies to patients undergoing CVS. This study aimed to determine the risk of developing PrU in patients undergoing CVS and assess its occurrence within 24 hours of surgery.

## METHOD

### Design of Study

The study had a descriptive and observational design.

### Sample Selection

Patients admitted to the CVS Ward for surgery between March and September 2022 in a public hospital located in the western region of Türkiye were included in the study. Patients over 18 years of age who were scheduled to undergo elective surgery without preoperative PrUs, and who agreed to participate in the study, were included. According to the G*Power 3.0.10 program, it was determined that 200 patients were sufficient for the sample with a small effect size of d = 0.2, 80% power, and 5% margin of error^(17)^.

### Data Collection Tools

Patient Introduction Form: This form was developed by researchers on the basis of the relevant literature^(16,18)^. The form included demographic variables (e.g. gender, age, Braden Scale score) of the patients and some other variables related to surgery (e.g. duration of stay on the heart-lung machine, length of surgery, some blood work values in the preoperative period (albumin, hemoglobin, hematocrit, and blood glucose level), use of support surfaces, blood pressure values during surgery, and development of PrU in the post-operative period.

3S IPIRAS: This scale was developed in 2015, and the Turkish validity and reliability study (Cronbach’s alpha 0.68) was conducted in 2018^(11)^. The scale assesses the risk factors for pressure injury in patients undergoing surgery (e.g. overall skin condition, activity status, height/weight ratio (body mass index (BMI)), amount of bleeding in surgery, body temperature, stress to the skin, duration of surgery, and the location of the surgery). Each item included in this scale is assessed and scored with numbers from one to four. The total scores that can be obtained from the scale range from 9 to 36. A score greater than 23 indicates the patient is at risk of developing a pressure injury in the OR. In the present study, Cronbach’s alpha coefficient was 0.714.

### Data Collection Procedure

The data were collected in three stages:

– Stage 1: The descriptive characteristics of the patient for the Patient Information Form, the Braden Scale score in the preoperative period, activity status, and the BMI were collected by face-to-face interview with the patient before surgery. Each interview lasted about 15 minutes, while the type of anesthesia and the blood work results were obtained from the patient’s records.
– Stage 2*:* Components of the 3S IPIRAS were completed by the first researcher using face-to-face interviews.
– Stage 3: Within 24 hours of the surgery, the pressure areas of the patients were evaluated by the first researcher during inspection using the staging system of the National PrU Advisory Panel (NPUAP)^(12)^. The patients’ primary nurse of the patients also evaluated their skin simultaneously. Each patient’s assessment lasted 10–15 minutes. After completing assessments, a joint decision was made to stage the PrUs. The skin’s condition was evaluated within 24 hours of the operation to identify the PrUs on the first post-operative day. All the patients are placed on a standard OR table covered with a matrix. A heating pad was placed between the operating table and the patient to control body temperature. In addition, a gel-filled bagel-shaped protective device was used under the patients’ heads.

### Data Analysis

Statistical analyses were performed using the IBM SPSS Statistics 24 package. Descriptive statistics were given in numbers and percentages. The independent samples t-test, ANOVA, the Mann-Whitney U test, and the Kruskal-Wallis H test were used to compare the measured values. Tukey or Tamhane tests, Bonferroni correction, Pearson, and Spearman correlations were used to analyze suitable data. The non-parametric tests were used for measurement values that did not show a normal distribution. The Mann-Whitney U test was used to compare the measurements obtained from two independent groups, and the Kruskal-Wallis H test was used to compare three or more independent groups. Bonferroni correction was used for bilateral comparisons of variables with significant differences in three or more groups. Pearson and Spearman correlations were used to examine the relationship between values^(17)^.

### Ethical Aspects

Permission to conduct the research was granted by the Clinical Research Ethics Committee of Bursa City Hospital at the Health Sciences University, Bursa, Türkiye (dated: Dec 29, 2021; protocol number: 2021-24/11).

## RESULTS

The patients’ mean age was 63.61 ± 9.14 (years), 65% were male, and 40.5% were overweight. Seventy-two-point-five percent (72.5%) of the patients had CABG surgery, and 98.5% received general anesthesia. Th characteristics of patients are presented in Table 1.

**Table 1:** Distribution of patients’ characteristics – Bursa, Türkiye, 2022.

Variables	Mean ± SD	Numbers (n = 200)	Percentage (%)
Age Group	63.61 ± 9.14 (years)		
<60 years		64	32.0
60–64 years		42	21.0
65–69 years		37	18.5
≥70 years		57	28.5
Gender			
Female		70	35.0
Male		130	65.0
BMI	26.68 ± 4.67kg/m^2^		
Normal (18.5–24.9 kg/m^2^)		45	22.5
Overweight (25.0–29.9 kg/m^2^)		81	40.5
Obese (≥30 kg/m^2^)		74	37.0
Smoking			
Yes		71	35.5
No		129	64.5
Diagnosis of DM			
Yes		99	49.5
No		101	50.5
Diagnosis of HT			
Yes		113	56.5
No		87	43.5
COPD			
Yes		17	8.5
No		183	91.5
History of MI			
Yes		108	54.0
No		92	46.0
Type of Surgery			
CABG		145	72.5
Heart Valve Surgery + CABG		20	10.0
Carotid endarterectomy		18	9.0
Other		17	8.5
Type of Anesthesia			
General		197	98.5
Spinal epidural		3	1.5
Pressure ulcer development after 24 hours of surgery			
Yes		28	14.0
No		172	86.0

The Braden Scale score was calculated as 14.03 ± 1.51 (range: 11–15), and the 3S IPIRAS score was 17.83 ± 2.15 (range: 11–24). The quantitative variables of the patients are presented in Table 2.

**Table 2:** Patients’ quantitative variables – Bursa, Türkiye, 2022.

Variables	Mean	Range	Median
Braden Scale score	14.03 ± 1.51	11–15	15.0
IPIRAS score	17.83 ± 2.15	11–24	18.0
Length of stay in the heart and lung machine (minutes)	101.54 ± 43.17	44–258	89.0
Length of surgery (minutes)	261.00 ± 75.10	90–570	240.0
Albumin	35.67 ± 5.29	20–61.3	35.9
Hemoglobin	12.86 ± 2.12	7.5–23.2	12.9
Glucose	147.75 ± 65.77	34.8–431	127.0
Hematocrit	37.21 ± 5.21	20.7–51.0	37.8
Systolic BP (min) mmHg	85.08 ± 13.58	50–140	80.0
Diastolic (min) mmHg	51.70 ± 10.32	30–100	50.0
Systolic (max) mmHg	145.65 ± 21.07	100–200	150.0
Diastolic (max) mmHg	77.70 ± 14.82	20–130	80.0

A comparison of the patients’ characteristics with 3S IPIRAS Scores is given in Table 3. Age, diabetes, hypertension (HT), Chronic Obstructive Pulmonary Disease (COPD), and history of myocardial infarction (MI) did not increase the risk of PrU development (*P* > 0.05). Gender increased the risk of PrU development in the OR (Z = −2.884; *P* = 0.004). BMI affected the risk of PrU development (χ^2^ = 55.371; *P* < 0.001). There was a significant difference between the scores of the patients according to their smoking status (Z = −2.797; *P* = 0.005): non-smokers’ scores were significantly higher than those of smokers (Table 3). A significant difference was found between those with CABG, valve surgery patients, and valve surgery + CABG patients (χ^2^ = 25.709; *P* < 0.001). The 3S IPIRAS scores of patients who underwent CABG, heart valve surgery, and valve surgery combined with CABG were significantly higher than those who had different surgeries (Table 3).

**Table 3:** Comparison of 3S IPIRAS scores with descriptive characteristics of patients – Bursa, Türkiye, 2022.

Variables	n = 200	3S IPIRAS score		Significance
		Mean ± SD	Median	
Age Group				
<60 years	64	17.75 ± 2.20	18.0 [2.0]	
60–64 years	42	17.81 ± 2.05	18.0 [2.0]	χ^2^ = 0.227
65–69 years	37	18.14 ± 1.76	18.0 [2.0]	p = 0.973
≥70 years	57	17.74 ± 2.40	18.0 [2.0]	
Gender				
Female	70	18.49 ± 2.13	18.0 [2.3]	Z = −2.884
Male	130	17.48 ± 2.07	17.0 [2.0]	**p = 0.004**
BMI				
Normal	45	16.36 ± 2.02	17.0 [1.5]	χ^2^ = 55,371
Overweight	81	17.68 ± 1.85	18.0 [1.0]	**p < 0.001**
Obese	74	18.89 ± 1.96	19.0 [2.0]	**[1–2,3] [2–3]**
Smoking				
Yes	71	17.31 ± 2.09	17.0 [3.0]	Z = −2.797
No	129	18.12 ± 2.13	18.0 [2.0]	**p = 0.005**
Diagnosis of DM				
Yes	99	17.89 ± 2.26	18.0 [2.0]	Z = −0.255
No	101	17.77 ± 2.04	18.0 [2.0]	p = 0.799
Diagnosis of HT				
Yes	113	17.96 ± 2.14	18.0 [2.0]	Z = −1.626
No	87	17.65 ± 2.16	18.0 [2.0]	p = 0.104
COPD				
Yes	17	18.41 ± 2.35	18.0 [3.0]	Z = −0.984
No	183	17.77 ± 2.13	18.0 [2.0]	p = 0.325
History of MI				
Yes	108	17.91 ± 1.85	18.0 [2.0]	Z = −0.198
No	92	17.74 ± 2.46	18.0 [2.0]	p = 0.843
Type of Surgery				
CABG	145	17.71 ± 1.84	18.0 [2.0]	χ^2^ = 25,709
Heart Valve Surgery + CABG	20	19.15 ± 1.42	19.0 [1.8]	**p < 0.001**
Carotid endarterectomy	18	19.39 ± 1.75	19.0 [2.0]	**[4–1,2,3]**
Other	17	15.64 ± 3.35	15.0 [6.0]	**[1–2,3]**
Pressure ulcer development after 24 hours of surgery				
Yes	28	19.21 ± 2.45	19.0 [3,0]	Z = −3.347
No	172	17.60 ± 2.01	18.0 [2,0]	**p < 0.001**

*Mann-Whitney U test (Z-table value) for comparing two independent groups in data that is not normally distributed; Kruskal-Wallis H” test (χ^2^-table value) statistics were used to compare three or more independent groups.

The 3S IPIRAS scores of patients who developed PrUs were significantly higher than those of patients with no PrUs (Z = −3.347; *P* < 0.001) (Table 3). A negative, weak, and statistically significant correlation was found between systolic-diastolic blood pressure (minimum) values and the 3S IPIRAS score (*P* < 0.05) (Table 4).

**Table 4:** Comparison of quantitative parameters of patients with 3S IPIRAS scores – Bursa, Türkiye, 2022.

Quantitative Variables Related to Patients (n = 200)	3S IPIRAS Score	
	r	*P*
Braden Scale score	−0.063	*P* = 0.377
Duration of use of heart and lung machine (minutes)	0.427	***P* < 0.001**
Length of surgery (minutes)	0.414	***P* < 0.001**
Albumin	−0.125	*P* = 0.078
Hemoglobin	0.001	*P* = 0.986
Glucose	0.122	*P* = 0.085
Hematocrit	0.053	*P* = 0.459
Systolic BP (min)mmHg	−0.233	***P* < 0.001**
Diastolic (min) mmHg	−0.204	***P* < 0.001**
Systolic (max) MmHg	−0.067	*P* = 0.349
Diastolic (max) mmHg	−0.029	*P* = 0.682

## DISCUSSION

This study aimed to determine the risk of PrUs in patients undergoing CVS and to assess their occurrence within 24 hours of the operation. The findings are discussed below with reference to the literature.

Increased age is a risk for PrU formation during surgery. In particular, being over the age of 70 triggers PrU development in cardiovascular surgery patients^(7)^. In the present study, age did not pose a risk with regard to developing PrUs. This result may be associated with the patients in the study being of relatively close. Being female gender was associated with the development of PrUs; the results of the present study are similar to those in the literature^(18)^. Another study emphasizes that males are in the higher-risk group^(6)^. Although age and gender pose important risks with regard to PrU development, one study indicated no relationship with these characteristics in CVS patients^(19)^. Obesity, that is, having a BMI of over 30 kg/m^2^, is a risk factor for PrUs^(19,20,21)^. Excess fat hinders tissue perfusion and being underweight can also be a risk due to the increased exposure of the patient’s bony prominences^(20)^. The present study found that being overweight and obese were serious risk factors for PrU development. Frequent habitual smoking notably increases the risk of PrU formation^(7,15)^, although the risk was only very slightly higher than in non-smokers in this study. We do not have any data on the frequency of smoking in this study, but nicotine does have a vasoconstrictor effect that affects the development of PrUs by decreasing oxygenation, which should not be overlooked^(15)^. Owing to the fact that smoking leads to chronic disease, it is also related to PrUs in the intraoperative period^(21)^. However, chronic diseases and a history of MI were not associated with PrU development in the present study, as in some other studies which have stated that chronic diseases are not an issue^(7,15)^.

The length of surgery is an important risk factor for the formation of PrUs, particularly surgeries lasting over four hours in the OR^(4,7,13,14,16)^. In the present study, the 3S IPIRAS scores of patients with heart valve surgery and heart valve surgery combined with CABG were significantly higher. This difference could be due to the length of the surgical procedure and insufficient oxygenation^(4,15,16)^.

Immobility decreases sensory perception, and inadequate lubrication and friction/tearing are preliminary stages in PrU development^(7)^. Insufficient support surfaces, moist skin, procedures lasting more than six hours, and the supine position required during CVS significantly increase the risk for PrUs^(7,16)^. A study conducted in patients undergoing CVS found that PrUs developed in 19.86% of them: among these patients, 87.88% of their PrU stages were classified as stage I, and 12.12% were rated as stage II^(7)^. Another study on surgical patients found that stage I PrUs were detected in 12.8% within 24 hours after surgery. The most affected areas were the buttocks, the elbow, the heel, the auricle, and the back^(18)^. In the present study, PrU was seen in 14.0% of the patients, and their 3S IPIRAS scores were higher prior to surgery. This result is similar to those of previous studies conducted on this topic. High-quality surfaces on OR tables may also help reduce PrU formation^(1,4)^. The NPUAP recommends that the OR table have a pressure-alternating support surface, such as a viscoelastic polymer pad^(12)^. Although the use of donut-shaped material prevents PrU development in open heart surgery patients^(19)^, the use of donut-shaped cushions is not recommended by the NPUAP^(12)^.

In open-heart surgery patients, interventions should be performed to prevent PrUs in the preoperative, preadmission, and perioperative periods^(14)^. The Braden risk scores of the patients in the present study determined that they were at moderate risk before their open-heart surgery. The 3S IPIRAS scores of these patients were as high as 17.83 ± 2.15. This result suggests that patients undergoing open heart surgery are at increased risk of developing PrUs, and that these patients require careful monitoring throughout the perioperative period.

According to the model developed to predict the occurrence of surgery-related PrUs in patients undergoing cardiovascular surgery, there are nine significant risks: preoperative hemoglobin level, serum sodium level, preoperative potassium level, prealbumin level, hypertension, smoking frequency, intraoperative mean temperature, minimum mean arterial pressure, and being over the age of 70^(16)^. Maintaining tissue oxygenation at an optimum level depends on blood pressure, and this is important when undergoing open heart surgery is important in preventing PrU formation^(7)^. Loss of tissue elasticity due to aging decreases in subcutaneous adipose tissue. Thus, low albumin levels and low muscle mass cause the skin to become more sensitive and increase the risk of PrUs^(6)^. Low hemoglobin, hematocrit, and albumin levels are essential^(4,7)^; albumin levels in particular have been associated with PrUs^(7,16,22)^_,_ although these findings are not compatible with the results of the present study. This outcome could be related to the time the laboratory work was carried out, which was usually five days before surgery. The systolic-diastolic blood pressure (minimum) values were a predisposing factor for PrUs in the present study, which is in accordance with other research^(7,18)^. Having a mean arterial pressure higher than >105 mm Hg has also been linked to device-related PrU formation in surgical patients^(5)^.

### Limitations

It is standard practice to use heating pads and gel bags in all patients undergoing CVS in the hospital where this study was conducted. No specific materials were available to place on the OR table for PrU prevention. During data collection in the post-operative period, observing the patients’ skin for PrU formation was difficult and time-consuming due to their unstable hemodynamic parameters.

## CONCLUSION

The results of this study show that patients undergoing cardiac surgery are at risk of developing PrUs, and that 14% of the patients developed a Stage-I PrU within 24 hours of the surgery. Taking better preventive measures throughout the perioperative period could help reduce this rate. Increasing awareness among healthcare staff is also essential in preventing PrUs. In addition, conducting longitudinal and randomized controlled studies with a focus on acquiring strong evidence could help highlight the incidence of PrUs in CVS patients. Further studies should thus be implemented with patients undergoing CVS.

## Data Availability

Data is available upon request by contacting the corresponding author.
