# Supplementary material for: Pressure ulcer risk in patients undergoing cardiovascular surgery and their occurrence within 24 hours of the operation
Source: Rev Esc Enferm USP. 2025 Aug 4;59:e20250081. doi: 10.1590/1980-220X-REEUSP-2025-0081en (PMC12323390; doi:10.1590/1980-220X-REEUSP-2025-0081en)
Supplement: Translation

# Translation: Riesgo de úlceras por presión en pacientes sometidos a cirugía cardiovascular y su aparición en las 24 horas siguientes a la operación

**DOI:** 10.1590/1980-220X-REEUSP-2025-0081es

**Authors:** Zeynep Gürer, Neriman Akansel, Nail Kahraman

**Affiliations:** 1 Health Sciences University, Bursa City Hospital, Operating Room Staff Nurse, Bursa, Türkiye.; 2 Bursa Uludag University, Faculty of Health Sciences, Department of Surgical Nursing, Bursa, Türkiye.; 3 Health Sciences University, Bursa City Hospital, Department of Cardiovascular Surgery, Bursa, Türkiye.

**Keywords:** Cirugía General, Atención de Enfermería, Quirófanos, Úlcera por Presión

## Abstract

**Objetivo:**

Este estudio tiene como objetivo determinar el riesgo de úlceras por presión (UPP) en pacientes sometidos a cirugía cardiovascular (CCV) y evaluar su aparición dentro de las 24 horas posteriores a la operación.

**Método:**

El estudio se realizó con pacientes de CCV (n = 200) que cumplían con los criterios de inclusión. Los datos se recopilaron mediante el Formulario de Introducción del Paciente y la Versión Turca de la Escala de Evaluación del Riesgo de Lesiones por Presión Intraoperatorias 3S (3S IPIRAS). El desarrollo de UPP se evaluó dentro de las 24 horas posteriores a la operación.

**Resultados:**

La edad promedio de los pacientes fue de 63,61 años y el 32,0% tenía menos de 60 años. El 65% de los pacientes eran hombres y el 40,5% tenía sobrepeso. El género femenino y el IMC influyeron en el riesgo de desarrollar UPP. Algunos pacientes (14%) desarrollaron una UPP de estadio I dentro de las 24 horas posteriores a la cirugía.

**Conclusión:**

Los pacientes sometidos a CCV tienen riesgo de desarrollar UPP, y la incidencia fue alta en este estudio. Son esenciales las medidas preventivas en el periodo perioperatorio para aumentar la concienciación entre el personal sanitario.

## INTRODUCCIÓN

Las úlceras por presión (UpP) son roturas localizadas que suelen encontrarse en prominencias óseas; están causadas por la exposición a presión externa sobre la piel o el tejido subcutáneo^(1)^. Los pacientes quirúrgicos corren el riesgo de desarrollar UpP a lo largo de todo el proceso perioperatorio, que incluye los cuidados preoperatorios, intraoperatorios y postoperatorios. Aunque los factores relacionados con el paciente (por ejemplo, las enfermedades crónicas, la edad, el sexo masculino, la obesidad, la malnutrición, el estado de la piel o la anemia) influyen en el desarrollo de las UpP, los incidentes relacionados con el entorno del quirófano (por ejemplo, la permanencia en la misma posición durante mucho tiempo, la duración de la intervención, la anestesia, el uso de vasopresores, la pérdida de sangre, la hipotermia, el equipo médico o la fricción) son innegables^(2,3,4,5,6)^. En cirugía cardiovascular (CCV) específicamente, los factores ambientales pueden provocar el desarrollo de UpP^(4,5)^. Las UpP relacionadas con dispositivos en protuberancias óseas, como la mejilla, el tórax, el talón y el pabellón auricular, son frecuentes en pacientes quirúrgicos^(5)^.

En un estudio, la incidencia de UpPs en pacientes adultos sometidos a cirugía cardiovascular fue del 19,86%^(7)^. Los porcentajes de UpPs relacionados con el proceso quirúrgico varían del 1,3% al 17,6%^(8,9)^. En un metaanálisis realizado para determinar la incidencia de UpPs en diferentes países, entre ellos Turquía, la incidencia llegó al 12%^(9)^. En otro estudio se detectaron lesiones por presión en estadio 1 en el 6,7% de los pacientes, y se observó que la aparición de UpP perjudicaba la calidad de la asistencia, amenazaba la seguridad de los pacientes, prolongaba la estancia hospitalaria y aumentaba el coste de la asistencia^(10)^. Sin embargo, es imposible eliminar por completo las UPR relacionadas con el quirófano debido a sus múltiples etiologías y a las dificultades para evaluar los factores de riesgo^(11)^.

La Guía de Referencia Rápida para la Prevención y el Tratamiento de la UpP (Prevention and Treatment Quick Reference Guideline) subraya que la evaluación del riesgo de UpP debe realizarse cada vez que cambie el estado del paciente, antes, durante y después de la intervención quirúrgica^(12)^. Aunque existen muchas escalas para evaluar el riesgo de UpP (la escala de Norton, la escala de Braden, la escala de Waterlow, la escala de Gosnell y la escala de Knoll), ninguna es capaz de predecir adecuadamente el riesgo de UpP en pacientes sometidos a cirugía. Un estudio realizado con la escala Munro PrU Risk Assessment Scale informó de que esta escala es eficaz para predecir las UpP en el postoperatorio en pacientes que han recibido anestesia general^(13)^. Los estudios controlados aleatorizados centrados en el proceso perioperatorio requieren un alto nivel de evidencia para la aparición de UpPs en pacientes con CCV^(14,15)^.

Se recomienda el uso de la Intraoperative Pressure Injury Risk Assessment Scale (Escala 3S de Evaluación del Riesgo de Lesiones por Presión Intraoperatoria 3S IPIRAS) para la evaluación de pacientes con alto riesgo de UpPs en el quirófano. En el estudio turco de validación de esta escala con varios pacientes quirúrgicos (n = 187), ocho pacientes evaluados con riesgo de desarrollar UpP en el quirófano experimentaron formación de UpP en estadio uno tras la cirugía^(11)^. El personal de enfermería quirúrgica es crucial para la seguridad del paciente a lo largo del proceso perioperatorio^(2)^. Las prácticas basadas en la evidencia y las intervenciones específicas para cada caso en el quirófano son esenciales para prevenir resultados indeseables como las UpP^(3,6,16)^.

La investigación sobre la evaluación del riesgo de UpP en el entorno del quirófano es limitada, y esto también se aplica a los pacientes sometidos a CCV. Este estudio tenía como objetivo determinar el riesgo de desarrollar UpP en pacientes sometidos a CCV y evaluar su aparición en las 24 horas siguientes a la cirugía.

## MÉTODO

### Diseño del Estudio

El estudio tenía un diseño descriptivo y observacional.

### Selección de la Muestra

Se incluyeron en el estudio los pacientes ingresados en la sala de CCV para cirugía entre marzo y septiembre de 2022 en un hospital público situado en la región occidental de Turquía. Se incluyeron pacientes mayores de 18 años que tenían programada una intervención quirúrgica electiva sin UpP preoperatoria y que aceptaron participar en el estudio. Según el programa G*Power 3.0.10, se determinó que 200 pacientes eran suficientes para la muestra con un tamaño del efecto pequeño de d = 0,2, una potencia del 80% y un margen de error del 5%^(17)^.

### Herramientas de Recolección de Datos

Formulario de presentación del paciente: Este formulario fue elaborado por los investigadores basándose en la bibliografía pertinente^(16,18)^. El formulario incluía variables demográficas (por ejemplo, sexo, edad, puntuación en la escala de Braden) de los pacientes y algunas otras variables relacionadas con la cirugía (por ejemplo, duración de la estancia en el sistema de circulación extracorpórea, duración de la cirugía, algunos valores de los análisis de sangre en el período preoperatorio (albúmina, hemoglobina, hematocrito y nivel de glucosa en sangre), uso de superficies de apoyo, valores de la presión arterial durante la cirugía y desarrollo de UpP en el período postoperatorio.

3S IPIRAS: Esta escala se desarrolló en 2015, y el estudio turco de validez y fiabilidad (alfa de Cronbach 0,68) se realizó en 2018^(11)^. La escala evalúa los factores de riesgo de lesiones por presión en pacientes sometidos a cirugía (por ejemplo, el estado general de la piel, el estado de actividad, la relación altura/peso (índice de masa corporal [IMC]), la cantidad de sangrado en la cirugía, la temperatura corporal, el estrés en la piel, la duración de la cirugía y el lugar de la cirugía). Cada ítem incluido en esta escala se evalúa y puntúa con números del uno al cuatro. Las puntuaciones totales que pueden obtenerse de la escala oscilan entre 9 y 36. Una puntuación superior a 23 indica que el paciente está en riesgo de desarrollar una lesión por presión en el quirófano. En el presente estudio, el coeficiente alfa de Cronbach fue de 0,714.

### Procedimiento de Recolección de Datos

Los datos se recogieron en tres etapas:

– Etapa 1: Las características descriptivas del paciente para el formulario de información del paciente, la puntuación de la escala de Braden en el periodo preoperatorio, el estado de actividad y el IMC se recogieron mediante entrevista cara a cara con el paciente antes de la intervención quirúrgica. Cada entrevista duró unos 15 minutos, mientras que el tipo de anestesia y los resultados de los análisis de sangre se obtuvieron del historial del paciente.
– Etapa 2*:* El primer investigador completó los componentes de las 3S IPIRAS mediante entrevistas presenciales.
– Etapa 3: A las 24 horas de la intervención quirúrgica, las zonas de presión de los pacientes fueron evaluadas por el primer investigador durante la inspección utilizando el sistema de estadificación del National PrU Advisory Panel (NPUAP)^(12)^. La enfermera de cabecera de los pacientes también evaluó su piel simultáneamente. La evaluación de cada paciente duró entre 10 y 15 minutos. Tras completar las evaluaciones, se tomó la decisión conjunta de estadificar las UpPs. El estado de la piel se evaluó a las 24 horas de la intervención para identificar las UpPs en el primer día postoperatorio. Todos los pacientes se colocaron en una mesa de quirófano estándar cubierta con una matriz. Se colocó una almohadilla térmica entre la mesa de operaciones y el paciente para controlar la temperatura corporal. Además, se utilizó un dispositivo protector en forma de rosquilla, relleno de gel bajo la cabeza de los pacientes.

### Análisis de Datos

Los análisis estadísticos se realizaron con el paquete IBM SPSS Statistics 24. Las estadísticas descriptivas se expresaron en cifras y porcentajes. Para comparar los valores medidos se utilizaron la prueba t de muestras independientes, ANOVA, la prueba U de Mann-Whitney y la prueba H de Kruskal-Wallis. Para analizar los datos adecuados se utilizaron las pruebas de Tukey o Tamhane, la corrección de Bonferroni y las correlaciones de Pearson y Spearman. Las pruebas no paramétricas se utilizaron para los valores medidos que no mostraban una distribución normal. Se utilizó la prueba U de Mann-Whitney para comparar las mediciones obtenidas de dos grupos independientes, y la prueba H de Kruskal-Wallis para comparar tres o más grupos independientes. Se utilizó la corrección de Bonferroni para las comparaciones bilaterales de variables con diferencias significativas en tres o más grupos. Se utilizaron correlaciones de Pearson y Spearman para examinar la relación entre los valores^(17)^.

### Aspectos Éticos

La autorización para llevar a cabo la investigación fue concedido por el Comité Ético de Investigación Clínica del Hospital de la Ciudad de Bursa en la Universidad de Ciencias de la Salud, Bursa (con fecha: 29 de diciembre de 2021; número de protocolo: 2021-24/11).

## RESULTADOS

La edad media de los pacientes era de 63,61 ± 9,14 (años), el 65% eran hombres y el 40,5% tenían sobrepeso. El 72,5% de los pacientes se sometieron a cirugía de revascularización coronaria y el 98,5% recibieron anestesia general. Las características de los pacientes se presentan en la Tabla 1.

**Tabla 1:** Distribución de las características de los pacientes – Bursa, Turquía, 2022.

Variables	Media ± DE	Números (n = 200)	Porcentaje (%)
Grupo de edad	63,61 ± 9,14 (años)		
<60 años		64	32.0
60–64 años		42	21.0
65–69 años		37	18.5
≥70 años		57	28.5
Género			
Mujer		70	35.0
Hombre		130	65.0
IMC	26,68 ± 4,67 kg/m^2^		
Normal (18,5–24,9 kg/m^2^)		45	22.5
Sobrepeso (25,0–29,9 kg/m^2^)		81	40.5
Obeso (≥30 kg/m^2^)		74	37.0
Fumar			
Sí		71	35.5
No		129	64.5
Diagnóstico de DM			
Sí		99	49.5
No		101	50.5
Diagnóstico de HTA			
Sí		113	56.5
No		87	43.5
EPOC			
Sí		17	8.5
No		183	91.5
Historia de IM			
Sí		108	54.0
No		92	46.0
Tipo de cirugía			
By-pass coronario		145	72.5
Cirugía de válvulas cardiacas + by-pass		20	10.0
Endarterectomía carotídea		18	9.0
Otros		17	8.5
Tipo de anestesia			
General		197	98.5
Epidural espinal		3	1.5
Desarrollo de úlceras por presión a las 24 horas de la intervención			
Sí		28	14.0
No		172	86.0

La puntuación de la escala de Braden se calculó en 14,03 ± 1,51 (rango: 11–15), y la puntuación 3S IPIRAS fue de 17,83 ± 2,15 (rango: 11–24). Las variables cuantitativas de los pacientes se presentan en la Tabla 2.

**Tabla 2:** Variables cuantitativas de los pacientes – Bursa, Turquía, 2022.

Variables	Media	Gama	Mediana
Puntuación en la escala de Braden	14.03 ± 1.51	11–15	15.0
Puntuación IPIRAS	17.83 ± 2.15	11–24	18.0
Duración de la estadía en la máquina cardiopulmonar (minutos)	101.54 ± 43.17	44–258	89.0
Duración de la intervención (minutos)	261.00 ± 75.10	90–570	240.0
Albúmina	35.67 ± 5.29	20–61.3	35.9
Hemoglobina	12.86 ± 2.12	7.5–23.2	12.9
Glucosa	147.75 ± 65.77	34.8–431	127.0
Hematocrito	37.21 ± 5.21	20.7–51.0	37.8
PA sistólica (min) mmHg	85.08 ± 13.58	50–140	80.0
Diastólica (min) mmHg	51.70 ± 10.32	30–100	50.0
Sistólica (máx) mmHg	145.65 ± 21.07	100–200	150.0
Diastólica (máx) mmHg	77.70 ± 14.82	20–130	80.0

En la Tabla 3 se comparan las características de los pacientes con las puntuaciones 3S IPIRAS. La edad, la diabetes, la hipertensión arterial (HTA), la enfermedad pulmonar obstructiva crónica (EPOC) y los antecedentes de infarto de miocardio (IM) no aumentaron el riesgo de desarrollo de UpP *(P* > 0,05). El sexo aumentó el riesgo de desarrollo de UpP en la OR (Z = −2,884; *P = 0*,004). El IMC afectó al riesgo de desarrollo de UpP (χ^2^ = 55,371; P < 0,001). Hubo una diferencia significativa entre las puntuaciones de los pacientes según su hábito tabáquico (Z = −2,797; *P = 0*,005): las puntuaciones de los no fumadores fueron significativamente superiores a las de los fumadores (Tabla 3). Se encontró una diferencia significativa entre los pacientes con By-pass, los pacientes con cirugía valvular y los pacientes con cirugía valvular + by-pass (χ^2^ = 25,709; *P < 0*,001). Las puntuaciones 3S IPIRAS de los pacientes sometidos a by-pass, cirugía valvular cardiaca y cirugía valvular combinada con by-pass fueron significativamente superiores a las de los sometidos a cirugías diferentes (Tabla 3).

**Tabla 3:** Comparación de las puntuaciones 3S IPIRAS con las características descriptivas de los pacientes – Bursa, Turquía, 2022.

Variables	n = 200	Puntuación 3S IPIRAS		Significado
		Media ± DE	Mediana	
Grupo de edad				
<60 años	64	17.75 ± 2.20	18.0 [2.0]	
60–64 años	42	17.81 ± 2.05	18.0 [2.0]	χ^2^ = 0.227
65–69 años	37	18.14 ± 1.76	18.0 [2.0]	p = 0.973
≥70 años	57	17.74 ± 2.40	18.0 [2.0]	
Género				
Mujer	70	18.49 ± 2.13	18.0 [2.3]	Z = −2.884
Hombre	130	17.48 ± 2.07	17.0 [2.0]	**p = 0.004**
IMC				
Normal	45	16.36 ± 2.02	17.0 [1.5]	χ^2^ = 55,371
Sobrepeso	81	17.68 ± 1.85	18.0 [1.0]	**p < 0.001**
Obesos	74	18.89 ± 1.96	19.0 [2.0]	**[1–2,3] [2–3]**
Fumar				
Sí	71	17.31 ± 2.09	17.0 [3.0]	Z = −2.797
No	129	18.12 ± 2.13	18.0 [2.0]	**p = 0.005**
Diagnóstico de DM				
Sí	99	17.89 ± 2.26	18.0 [2.0]	Z = −0.255
No	101	17.77 ± 2.04	18.0 [2.0]	p = 0.799
Diagnóstico de HTA				
Sí	113	17.96 ± 2.14	18.0 [2.0]	Z = −1.626
No	87	17.65 ± 2.16	18.0 [2.0]	p = 0.104
EPOC				
Sí	17	18.41 ± 2.35	18.0 [3.0]	Z = −0.984
No	183	17.77 ± 2.13	18.0 [2.0]	p = 0.325
Historia de IM				
Sí	108	17.91 ± 1.85	18.0 [2.0]	Z = −0.198
No	92	17.74 ± 2.46	18.0 [2.0]	p = 0.843
Tipo de cirugía				
By-pass	145	17.71 ± 1.84	18.0 [2.0]	χ^2^ = 25,709
Cirugía de válvulas cardiacas + By-pass	20	19.15 ± 1.42	19.0 [1.8]	**p < 0.001**
Endarterectomía carotídea	18	19.39 ± 1.75	19.0 [2.0]	**[4–1,2,3]**
Otros	17	15.64 ± 3.35	15.0 [6.0]	**[1–2,3]**
Desarrollo de úlceras por presión a las 24 horas de la intervención				
Sí	28	19.21 ± 2.45	19.0 [3,0]	Z = −3.347
No	172	17.60 ± 2.01	18.0 [2,0]	**p < 0.001**

*Se utilizó la prueba U de Mann-Whitney (valor de la tabla Z) para comparar dos grupos independientes en datos que no tienen una distribución normal; la prueba H” de Kruskal-Wallis (valor de la tabla χ^2^) para comparar tres o más grupos independientes.

Las puntuaciones 3S IPIRAS de los pacientes que desarrollaron UpPs fueron significativamente superiores a las de los pacientes sin UpPs (Z = −3,347; *P* < *0*,001) (Tabla 3). Se encontró una correlación negativa, débil y estadísticamente significativa entre los valores de presión arterial sistólica-diastólica (mínima) y la puntuación 3S IPIRAS *(P* < *0*,05) (Tabla 4).

**Tabla 4:** Comparación de parámetros cuantitativos de pacientes con puntuaciones 3S IPIRAS – Bursa, Turquía, 2022.

Variables cuantitativas relacionadas con los pacientes (n = 200)	Puntuación 3S IPIRAS	
	r	*P*
Puntuación en la escala de Braden	−0.063	*P* = 0.377
Duración del uso de la máquina cardiopulmonar (minutos)	0.427	***P* < 0.001**
Duración de la intervención (minutos)	0.414	***P* < 0.001**
Albúmina	−0.125	*P* = 0.078
Hemoglobina	0.001	*P* = 0.986
Glucosa	0.122	*P* = 0.085
Hematocrito	0.053	*P* = 0.459
PA sistólica (min) mmHg	−0.233	***P* < 0.001**
Diastólica (min) mmHg	−0.204	***P* < 0.001**
Sistólica (máx) mmHg	−0.067	*P* = 0.349
Diastólica (máx) mmHg	−0.029	*P* = 0.682

## DISCUSIÓN

El objetivo de este estudio era determinar el riesgo de UpPs en pacientes sometidos a CCV y evaluar su aparición en las 24 horas siguientes a la operación. Los resultados se discuten a continuación con referencia a la bibliografía.

El aumento de la edad es un riesgo para la formación de UpP durante la cirugía. En particular, ser mayor de 70 años desencadena el desarrollo de UpP en pacientes sometidos a cirugía cardiovascular^(7)^. En el presente estudio, la edad no supuso un riesgo con respecto al desarrollo de UpP. Este resultado puede estar asociado a que los pacientes del estudio eran de edad relativamente próxima. El sexo femenino se asoció con el desarrollo de UpPs; los resultados del presente estudio son similares a los de la literatura^(18)^. Otro estudio destaca que los hombres pertenecen al grupo de mayor riesgo^(6)^. Aunque la edad y el sexo suponen riesgos importantes con respecto al desarrollo de UpP, un estudio no indicó ninguna relación con estas características en pacientes con CCV^(19)^. La obesidad, es decir, tener un IMC superior a 30 kg/m^2^, es un factor de riesgo de UpP^(19–21)^. El exceso de grasa dificulta la perfusión tisular y tener un peso inferior al normal también puede ser un riesgo debido a la mayor exposición de las prominencias óseas del paciente^(20)^. En el presente estudio se observó que el sobrepeso y la obesidad eran factores de riesgo graves para el desarrollo de UpP. El tabaquismo habitual frecuente aumenta notablemente el riesgo de formación de UpP^(7,15)^, aunque en este estudio el riesgo era sólo muy ligeramente superior al de los no fumadores. No disponemos de datos sobre la frecuencia del tabaquismo en este estudio, pero la nicotina tiene un efecto vasoconstrictor que afecta al desarrollo de las UpPs al disminuir la oxigenación, lo que no debe pasarse por alto^(15)^. Debido a que el tabaquismo conduce a enfermedades crónicas, también está relacionado con las UpPs en el periodo intraoperatorio^(21)^. Sin embargo, las enfermedades crónicas y los antecedentes de IM no se asociaron con el desarrollo de UpP en el presente estudio, como en algunos otros estudios que han afirmado que las enfermedades crónicas no son un problema^(7,15)^.

La duración de la intervención quirúrgica es un factor de riesgo importante para la formación de UpPs, en particular las cirugías que duran más de cuatro horas en el quirófano^(4,7,13,14,16)^. En el presente estudio, las puntuaciones 3S IPIRAS de los pacientes con cirugía valvular cardiaca y cirugía valvular cardiaca combinada con CABG fueron significativamente superiores. Esta diferencia podría deberse a la duración de la intervención quirúrgica y a una oxigenación insuficiente^(4,15,16).^

La inmovilidad disminuye la percepción sensorial, y la lubricación inadecuada y la fricción/desgarro son etapas preliminares en el desarrollo de UpP^(7)^. Las superficies de apoyo insuficientes, la piel húmeda, los procedimientos que duran más de seis horas y la posición supina requerida durante la CCV aumentan significativamente el riesgo de UpP^(7,16)^. En un estudio realizado en pacientes sometidos a una CCV se observó que el 19,86% de ellos presentaban UDP: el 87,88% de estos pacientes se encontraban en el estadio I de la UDP y el 12,12% en el estadio II ^(7)^. En otro estudio sobre pacientes quirúrgicos se observó que en el 12,8% de los casos se detectaba UpP en estadio I en las 24 horas siguientes a la intervención quirúrgica. Las zonas más afectadas fueron las nalgas, el codo, el talón, el pabellón auricular y la espalda^(18)^. En el presente estudio, se observó UpP en el 14,0% de los pacientes, y sus puntuaciones 3S IPIRAS eran más altas antes de la cirugía. Este resultado es similar al de estudios anteriores realizados sobre este tema. Las superficies de alta calidad de las mesas de quirófano también pueden ayudar a reducir la formación de UpP^(1,4)^. La NPUAP recomienda que la mesa de quirófano tenga una superficie de apoyo que altere la presión, como una almohadilla de polímero viscoelástico ^(12)^. Aunque el uso de material en forma de rosquilla previene el desarrollo de UpP en pacientes de cirugía a corazón abierto^(19)^, la NPUAP no recomienda el uso de cojines en forma de rosquilla^(12)^.

En los pacientes sometidos a cirugía a corazón abierto, deben realizarse intervenciones para prevenir las UpP en los periodos preoperatorio, previo al ingreso y perioperatorio^(14)^. Las puntuaciones de riesgo Braden de los pacientes del presente estudio determinaron que presentaban un riesgo moderado antes de la intervención a corazón abierto. Las puntuaciones 3S IPIRAS de estos pacientes fueron tan altas como 17,83 ± 2,15. Este resultado sugiere que los pacientes sometidos a cirugía a corazón abierto tienen un mayor riesgo de desarrollar UpPs, y que estos pacientes requieren un seguimiento cuidadoso durante todo el período perioperatorio.

Según el modelo desarrollado para predecir la aparición de UpP relacionadas con la cirugía en pacientes sometidos a cirugía cardiovascular, existen nueve riesgos significativos: nivel de hemoglobina preoperatoria, nivel de sodio sérico, nivel de potasio preoperatorio, nivel de prealbúmina, hipertensión, frecuencia de tabaquismo, temperatura media intraoperatoria, presión arterial media mínima y tener más de 70 años^(16)^. Mantener la oxigenación tisular en un nivel óptimo depende de la presión arterial, y esto es importante cuando se somete a una intervención a corazón abierto es importante para prevenir la formación de UpP^(7)^. La pérdida de elasticidad tisular debida al envejecimiento disminuye en el tejido adiposo subcutáneo. Por lo tanto, los niveles bajos de albúmina y la escasa masa muscular hacen que la piel se vuelva más sensible y aumentan el riesgo de UpPs^(6)^. Los niveles bajos de hemoglobina, hematocrito y albúmina son esenciales^(4,7)^; los niveles de albúmina en particular se han asociado con UpPs^(7,16,22)^ aunque estos hallazgos no son compatibles con los resultados del presente estudio. Este resultado podría estar relacionado con el momento en que se realizó la analítica, que normalmente fue cinco días antes de la cirugía. Los valores de presión arterial sistólica-diastólica (mínima) fueron un factor predisponente de UpPs en el presente estudio, lo que concuerda con otras investigaciones^(7,18)^. Tener una presión arterial media superior a >105 mm Hg también se ha relacionado con la formación de UpP relacionadas con dispositivos en pacientes quirúrgicos^(5)^.

### Limitaciones

En el hospital donde se realizó este estudio es práctica habitual utilizar almohadillas térmicas y bolsas de gel en todas las pacientes que se someten a una CCV. No se disponía de materiales específicos para colocar en la mesa de quirófano para la prevención de la UpP. Durante la recogida de datos en el periodo postoperatorio, la observación de la piel de los pacientes para detectar la formación de UpP resultó difícil y requirió mucho tiempo debido a la inestabilidad de sus parámetros hemodinámicos.

## CONCLUSIÓN

Los resultados de este estudio muestran que los pacientes sometidos a cirugía cardiaca corren el riesgo de desarrollar UpP, y que el 14% de los pacientes desarrollaron una UpP de estadio I en las 24 horas siguientes a la intervención. La adopción de mejores medidas preventivas durante todo el periodo perioperatorio podría ayudar a reducir esta tasa. La concienciación del personal sanitario también es esencial para prevenir las UpPs. Además, la realización de estudios longitudinales y aleatorizados controlados centrados en la adquisición de evidencias más fuertes, podría ayudar a poner de relieve la incidencia de las UpPs en pacientes sometidos a CCV. Así pues, deberían realizarse más estudios con pacientes sometidos a CCV.

## Data Availability

Los datos están disponibles a pedido contactando al autor correspondiente.
